# Behavioral Fatigue: Real Phenomenon, Naïve Construct, or Policy Contrivance?

**DOI:** 10.3389/fpsyg.2020.589892

**Published:** 2020-11-05

**Authors:** Nigel Harvey

**Affiliations:** Department of Experimental Psychology, University College London, London, United Kingdom

**Keywords:** Covid-19, government policy, behavioral fatigue, advisors, mitigation

## Abstract

In some countries, government policies to combat Covid-19 have been based on the notion that behavioral fatigue prevents people maintaining self-isolation and other restrictions to their life styles for more than a short time. By 16 March 2020, 681 United Kingdom behavioral scientists had signed an open letter to their government asking it to reveal the evidence that shows that behavioral fatigue exists. Nothing was forthcoming. The provenance of concept remains a mystery but modelers have argued that the delay in implementing lockdown policies, for which it was at least partly responsible, led to the loss of at least 20,000 lives. Here, I consider whether behavioral fatigue is a real phenomenon by assessing (a) direct evidence consistent and inconsistent with its existence and (b) indirect evidence drawn from other domains. I conclude that evidence for it is not sufficient to constrain policy. It is reasonable to conclude that behavioral fatigue is either a naïve construct or a myth that arose during the development of policy designed to tackle the Covid-19 crisis.

## Introduction

There are two approaches to dealing with disease transmission: suppression and mitigation. Suppression requires the reproduction number, *R* (the average number of secondary cases each case generates), be reduced below 1.0 *to lower the number* of infected people. Mitigation merely requires that *R* reduced (without bringing it below 1.0) *to lower the rate of increase in the number* of infected people. Until 16 March 2020, the government in the United Kingdom, unlike those in most other countries, favored mitigation. There were two arguments for this: First, building up herd immunity to reduce transmission requires about 60% of the population to become infected; second, there was a concern that the population would comply with measures needed for suppression only for a short time because of behavioral fatigue.

The first argument collapsed when modeling showed that producing herd immunity would result in about 250,000 deaths and a demand for critical care that the health service could not meet (Ferguson et al., 2020). However, the switch to a suppression policy on 16 March increased concern about effects of behavioral fatigue. Here, I document that concern and assess whether it has a sound basis.

## Behavioral Fatigue: Provenance of the Concept

At a United Kingdom government press conference on 9 March, Professor Chris Whitty, the United Kingdom Chief Medical Officer, argued that it was too soon to implement a lockdown: “There is a risk that if we go too early, people will understandably get fatigued and it will be difficult to sustain this over time.”^1^ At another such press conference on 12 March, Sir Patrick Vallance, the United Kingdom Chief Scientific Adviser, said that, if you tell people to stay at home too early, they get fed up with this at the very point that you need them to stay at home. “Anything too onerous suggested by the government … might be adopted enthusiastically for a few weeks but then people get bored and leave their homes just as the peak of the illness hits, the government fears” (Proctor, 2020). It appears that both government officers had received the same advice.

Where did this advice come from? Members of the United Kingdom government’s Scientific Advisory Group on Emergencies (SAGE) and the Scientific Pandemic Influenza Group on Behavioral Science (SPI-B) that feeds its advice into SAGE have said that they were not the source of the advice but SAGE minutes for 13 March 2020^2^ state that: “There is some evidence that people find quarantining harder to comply with the longer it goes on. The evidence is not strong but the effect is intuitive. There is no comparable evidence for social distancing measures, but experience suggests it is harder to comply with a challenging behaviour over a long period than over a short period.” Where did SAGE obtain the information on which this statement is based?

An interview with David Halpern, leader of the government’s Behavioral Insights Team (the “nudge unit”), strongly implied that he was the source of it (Hutton, 2020). According to Sodha (2020a), it was clear from this briefing “that he favoured delaying a lockdown because of the risk of ‘behavioural fatigue’, the idea that people will stick with restrictions for only so long, making it better to save social distancing for when more people are infected.” Because of Halpern’s involvement, his recommendations about the need to avoid “behavioural fatigue” were seen as “nudges,” even though they would not be categorized as such by Thaler and Sunstein (2008). Later, the Behavioural Insights Team released a statement saying that: “As it happens, the concept (of behavioural fatigue) did not come from BIT or our work, nor from that of SPI-B, the group of psychologists and social scientists who contribute advice to the UK’s Scientific Advisory Group on Emergencies” (Halpern and Harper, 2020).^3^

So where did the advice come from? According to Conn et al. (2020), “one senior Whitehall source said Whitty himself was the main advocate of the ‘fatigue’ notion, based partly on his own experience of patients in medical practice who do not see drug prescriptions through to their completion. A Downing Street spokesperson, responding on behalf of Whitty, emphasised that he was indeed concerned about timing interventions, and their impact on people’s wellbeing if introduced too early, and that Sage had agreed that a balance needed to be struck between the impact of measures, and the time the public could feasibly sustain them.” To some, this might appear to be an exercise in blame-shifting^4^ (Parker et al., 2020). It left others mystified: “I looked at where this pseudo-scientific idea of ‘behavioural fatigue’ came from. None of those I interviewed – including those on the behavioural science subcommittee of the emergency advisory group, Sage – knew” (Sodha, 2020b).

At the time of writing, no individual, advisory group, or government department has admitted that they were the source of the “behavioural fatigue” concept. This is perhaps not surprising given the effects of the lockdown delay produced by concerns about behavioral fatigue: Professor Neil Ferguson has estimated that introducing lockdown just 1 week earlier would have saved 20,000 lives (Stewart and Sample, 2020).

## Behavioral Fatigue: The Response From Behavioral Scientists

On 16 March 2020, 681 United Kingdom behavioral scientists (including the author) had signed an open letter to the government^5^:

“*We are writing as behavioural scientists to express concern about the timing of UK delay measures involving social distancing. … While we fully support an evidence-based approach to policy that draws on behavioural science, we are not convinced that enough is known about ‘behavioural fatigue’ or to what extent these insights apply to the current exceptional circumstances. Such evidence is necessary if we are to base a high-risk public health strategy on it. In fact, it seems likely that even those essential behaviour changes that are presently required (e.g., handwashing) will receive far greater uptake the more urgent the situation is perceived to be. ‘Carrying on as normal’ for as long as possible undercuts that urgency. … If ‘behavioural fatigue’ truly represents a key factor in the government’s decision to delay high-visibility interventions, we urge the government to share an adequate evidence base in support of that decision. If one is lacking, we urge the government to reconsider these decisions*.”

Given that concern about behavioral fatigue appears to have been a primary determinant of the government’s decision to mitigate rather that suppress infection caused by the virus, it is worth trying to address the issues that prompted this letter.

## Behavioral Fatigue: What Type of Concept is it?

Behavioral fatigue could be a real phenomenon. The term could refer to any one of a collection of factors that, over time, acts to reduce compliance with regulations (Bell, 2020). A few examples must suffice: (1) People may become more irritated with regulations the longer they have to abide by them and eventually make a decision to no longer to comply with them, (2) the degree to which people miss seeing their friends and taking part in social activities may increase over time and lead to reduced compliance, (3) people may become increasingly susceptible to those in their social circle who advocate a libertarian ideology that interprets government restriction on individual freedoms as something to be avoided, or (4) people may, perhaps because of reduced coverage in the media, falsely judge that risk of infection has decreased and so consider compliance with restrictions is less important than before. In my view, these are not cases of behavioral fatigue but rather putative phenomena that need to be distinguished from behavioral fatigue.

Alternatively, the term “behavioural fatigue” could refer to an underlying psychological mechanism that decreases people’s ability to behave in a certain way as a function of the amount of time that they have already been continuously behaving in that way. In other words, we should think of it as directly analogous to muscular fatigue. For example, we would expect people to recover from it after an interval in which the behavior is not performed and that the interval needed for recovery is greater when the behavior has been more intense or longer lasting. We would also expect it be associated with a feeling of tiredness or exhaustion. If behavioral fatigue is a real phenomenon, these are the types of characteristics we should expect it to have.

However, behavioral fatigue may not be a real phenomenon. It may be a naïve construct or, as Michie and West (2020, p. 1) term it, a “common-sense idea” that has “no basis in behavioural science.” Ontologically, this places behavioral fatigue within lay psychology (Furnham, 1988): Just as people have mistaken ideas about how the world works (Reiner et al., 2000), so they have mistaken ideas about factors that influence people’s behavior.

Finally, behavioral fatigue may be neither a real phenomenon nor a naïve construct. Italy went into lockdown on 9 March and most other Western European countries very soon after. The United Kingdom resisted this move until 23 March. This delay in imposing a lockdown has been attributed to the United Kingdom Prime Minister’s libertarian views (Tominey, 2020). If these views were indeed the true reason for not imposing a lockdown, policy makers may have felt the need to provide a separate rationale for this decision that they judged would be more acceptable to the general public. Hence, according to Michie and West (2020, p. 1), behavioral fatigue “was invoked in the UK as a justification of the catastrophic delay of strict social distancing measures.” In other words, behavioral fatigue was not the *reason* for the delay but was devised as a *post-hoc justification* for it. According to this account, the concept of behavioral fatigue is a myth contrived by policy makers in order to provide a *post-hoc* rationale for a decision that was actually made for quite different reasons. Burnham (1943, p. 269) argued: “The political life of the masses and the cohesion of society demand the acceptance of myths. A scientific attitude towards society does not permit belief in the truth of myths. But the leaders must profess, indeed foster, belief in the myths, or the fabric of society will crack and they be overthrown. In short, the leaders, if they themselves are scientific, must lie.”^6^

My aim, here, is to assess whether there is sufficient evidence to support the view that behavioral fatigue is a real phenomenon in the sense outlined above (i.e., a mechanism analogous to muscular fatigue rather than one without that quality but still able to explain reduced compliance over time). An absence of any clear evidence for behavioral fatigue in the current literature would suggest that whoever first developed the concept either misunderstood other research and used it to support their “common-sense idea” that such fatigue does exist or else decided that government policy was best served by promulgating the myth that it exists. Distinguishing between these latter two possibilities is not possible by searching the literature: It would have to await a future parliamentary or other inquiry into how the crisis has been handled by the United Kingdom government.

## Behavioral Fatigue: A Real Phenomenon?

In an interview (Devlin, 2020), Susan Michie, a member of the United Kingdom government’s SPI-B, said that the behavioral assumptions underlying the government’s Covid-19 policies were, in part, based on studies of human behaviur during past pandemics. A search of literature in April 2020 on the 2009 H1N1 influenza pandemic, the 2003 severe acute respiratory syndrome pandemic (SARS), and the current pandemic, initially using reviews (e.g., Bish and Michie, 2010; Brooks et al., 2020; Lunn et al., 2020) and later following up with searches referring to individual pandemics and the terms “behavioural,” “preventative measures,” and “fatigue,” yielded a number of studies potentially relevant to the issue of whether behavioral fatigue affects people’s responses to preventative measures.

Cowling et al. (2010) carried out 13 surveys of Hong Kong residents between April and November 2009 during the first wave of the 2009 H1N1 influenza pandemic. Results obtained from between 504 and 1,404 respondents showed that, as the epidemic grew, use of hygiene measures (e.g., face masks) remained fairly stable but that social distancing significantly declined. At first glance, this appears to be evidence of behavioral fatigue. However, another finding from Cowling et al. (2010) study was that people were more worried about being infected early in the outbreak and this, like social distancing, gradually declined over the study period. This implies that social distancing may have declined because people became less worried about being infected rather than because they were fatigued from abiding by the regulations.

Two studies indicate that periods of quarantine can have long-term effects (Lunn et al., 2020). Neither is easy to reconcile with the notion that behavioral fatigue reduces compliance with social distancing and hygiene measures. Cava et al. (2005, p. 402) interviewed 21 Canadians who, during the 2003 SARS outbreak, had been required to self-isolate for 10 days, receive no visitors, sleep alone, wear masks, and not share food or personal items. They found that: “Some participants stayed in quarantine past their release date … and described behavioral changes such as vigilant hand washing and avoiding crowds after the quarantine period.” This is the opposite of what would be predicted by behavioral fatigue.

Marjanovic et al. (2007) reported a study of 333 Canadian nurses who been placed in quarantine during the 2003 SARS epidemic. They found that engagement in avoidance behaviors (e.g., minimizing direct contact with patients, missing work, and refusing patient assignments) in 2004 was positively correlated with the time spent in quarantine in 2003. The longer they had spent avoiding certain behaviors in quarantine in 2003, the more avoidance behaviors they engaged in the following year. If we consider the nurses’ avoidance behavior scores as a measures of their avoidance of social contact, this is, again, the opposite of what would be predicted by behavioral fatigue; it is, instead, more consistent with habit development or with people perceiving measures to be more important when they are imposed for a longer period. However, if we consider nurses’ avoidance behavior to reflect other factors, such as lower work motivation, the study has no relevance to our current concerns.

This review suggests that *direct* evidence for (or against) the notion that people suffer from behavioral fatigue when complying with lockdown measures during epidemics is not currently sufficient to constrain policy. There are, however, other phenomena that government policy makers and their advisors may have seen as sufficiently relevant to the current situation to provide a scientific basis for their development of the notion of behavioral fatigue. I consider these next.

## Behavioral Fatigue: Extrapolation From Other Phenomena?

Various phenomena in other domains may have been identified by policy makers or their advisors as indicative of behavioral fatigue.

### Lack of Adherence to Medication

As we have seen, a “senior Whitehall source” attributed the introduction of the idea of behavioral fatigue into Covid-19 policy making to the United Kingdom Chief Medical Officer’s experience of his patients’ failure to adhere to their prescribed medicines (Conn et al., 2020). Failure to adhere to medication is certainly a major problem, particularly for those with chronic diseases. There are many reasons for it, including forgetting to take doses, lack of understanding that the medicine still needs to be taken when symptoms are absent, lack of information given to caregivers, and failure in doctor-patient communication (Kvarnström et al., 2018). However, there is no evidence that patients do not abide by their drug regimen because they have been fatigued by it.

### Ego-Depletion

One possibility is that behavioral fatigue results from ego-depletion. This is the idea that self-control is akin to a muscle that can become fatigued (Baumeister et al., 1998; Baumeister, 2002). Thus, if people need self-control to abide by government instructions to self-isolate, they may become fatigued because the resources needed for that self-control become depleted. However, large-scale attempts to replicate the findings on which the theory of ego-depletion is based have failed (Hagger et al., 2016) and meta-analyses have cast doubt on whether the phenomenon exists (Carter et al., 2015). Though the issue is far from settled, putative ego-depletion does not provide a sound basis for policy.

### Evacuation Fatigue

Research into behavioral responses to pandemics is part of disaster science (McNutt, 2015). This discipline also covers responses to earthquakes, tsunamis, volcanic eruptions, hurricanes, mudslides, wildfires, and other catastrophes. Its aim is to develop a coherent approach that allows knowledge to be accumulated so that what is learnt within one disaster domain can be usefully applied to others. It is serviced by international agencies, such as the United Nations Office for Disaster Risk Reduction,^7^ academic journals, such as *Progress in Disaster Science*, and research institutes, such as the Centre for Natural Hazards and Disaster Science^8^ and the Institute of Risk and Disaster Reduction.^9^

Catastrophic events can often be predicted, albeit with considerable uncertainty. This allows time for vulnerable populations to be evacuated. Often, however, the event does not occur and the population returns. Sometime later, they may be asked to evacuate again. There are many reports that compliance declines: people die because, after several false alarms, they develop “evacuation fatigue,” an effect that has been reported for a variety of disaster types, including wildfires (e.g., Metz, 2019), hurricanes (e.g., Childs, 2019), and mudslides (e.g., Biasotti et al., 2018).

Evacuation fatigue may genuinely be a type of fatigue: “(T)he task of executing a survival plan … is an extremely exhausting experience. Even those who planned well and made it out alive or sheltered in place from any catastrophic disaster later succumbed to the sheer fatigue of the event” (Woods, 2019). Alternatively, it may be the result of a more rational calculation. Each successive false alarm may signal to people that the evacuation order indicates that the probability of a catastrophe is not as high as they had previously thought: as a result, a time will come at which the expected cost of evacuating no longer exceeds the expected cost of not doing so. This is the well-known cry-wolf effect (Dow and Cutter, 1998; LeClerc and Joslyn, 2015).

Evacuation and quarantine have much in common. They both limit day-to-day activities, incur financial, emotional and other costs, are imposed by state or regional authorities, and last for durations that either are indefinite or, if not, are extendable. They are both disruptive and take away control that people have over their lives. It is not unreasonable to assume that reactions to them will be similar: If evacuations produce behavioral fatigue, quarantine and other anti-pandemic measures are also likely to produce it. However, this extrapolation, though possibly appealing to policy makers, is not legitimate. Evacuation does not change the probability of hurricanes, mudslides, wildfires, and other such catastrophes occurring but quarantine can reduce rates of infection. People realizing this are more likely to remain compliant than those facing repeated evacuation demands.

## Impact of Behavioral Fatigue on Current Policy

Though the United Kingdom government changed its Covid-19 policy from mitigation to suppression on 16 March 2020, ministers and their advisors remained concerned about potential effects of behavioral fatigue. For example, Ferguson et al. (2020) say that, because suppression policies that are continuous may need to be maintained for many months, an adaptive policy could be applied instead: Measures would be dropped when the number of ICU patients falls below an “off” threshold but re-introduced when they rise again above an “on” threshold. The assumption, here, is that this would avoid the behavioral fatigue assumed to arise with a continuous policy. However, it is possible that people would be less likely to comply with a re-introduced policy than to continue to comply with a continuous policy. If this proved to be the case, it would represent clear evidence against behavioral fatigue because people recover from fatigue after a break.

I have focused on developments within the United Kingdom but what I have said also has relevance to Sweden. That country maintained a mitigation policy based on the same assumptions about herd immunity and behavioral fatigue that governed United Kingdom policy before 16 March: In an interview (Orange, 2020), their state epidemiologist stated that he believed that it would be counterproductive to bring in the tightest restrictions at too early a stage: “I do not see any big reason to take measures that you can only keep up for a very limited amount of time.” Other European countries that did not delay attempts to suppress the pandemic had no need to resort to such arguments.

## Summary

Behavioral fatigue has been an important element in designing policies to counteract the Covid-19 pandemic and still is. However, there is little evidence that it exists or that it affects compliance with measures taken to reduce infection rates. Indeed, there have been many reports that the majority of people are reluctant to leave lockdown to use public transport, go to a pub or restaurant, or to attend sporting or other public events (e.g., Lister, 2020). Behavioral fatigue is not a real phenomenon: it must be either a naïve construct or a policy contrivance.

## Author Contributions

The author confirms being the sole contributor of this work and has approved it for publication.

### Conflict of Interest

The author declares that the research was conducted in the absence of any commercial or financial relationships that could be construed as a potential conflict of interest.

## Footnotes

^1^https://www.youtube.com/watch?v=Yc1alOEjDVA

^2^www.gov.uk/government/publications/sage-minutes-coronavirus-covid-19-response-13-march-2020

^3^It is still possible that Halpern himself provided the advice personally rather than as a BIT member.

^4^It is noticeable that Whitty himself did not confirm that he was the originator of “behavioural fatigue” but that “senior Whitehall sources” and a “Downing Street spokesperson” felt the need to speak for him.

^5^https://sites.google.com/view/covidopenletter/home

^6^Other policy-driven psychological myths include core, generic, or transferable skills, invented by the Manpower Services Commission in the 1970s to satisfy needs of employers despite research showing “there is little evidence that such general intertask transfer effects are possible” (Schmidt, 1975, p 61).

^7^www.undrr.org

^8^www.cnds.se

^9^www.ucl.ac.uk/risk-disaster-reduction
